# *De novo* detection of differentially bound regions for ChIP-seq data using peaks and windows: controlling error rates correctly

**DOI:** 10.1093/nar/gku351

**Published:** 2014-05-22

**Authors:** Aaron T.L. Lun, Gordon K. Smyth

**Affiliations:** 1 The Walter and Eliza Hall Institute of Medical Research, 1G Royal Parade, Parkville, VIC 3052, Australia; 2 Department of Medical Biology, The University of Melbourne, Parkville, VIC 3010, Australia; 3 Department of Mathematics and Statistics, The University of Melbourne, Parkville, VIC 3010, Australia

## Abstract

A common aim in ChIP-seq experiments is to identify changes in protein binding patterns between conditions, i.e. differential binding. A number of peak- and window-based strategies have been developed to detect differential binding when the regions of interest are not known in advance. However, careful consideration of error control is needed when applying these methods. Peak-based approaches use the same data set to define peaks and to detect differential binding. Done improperly, this can result in loss of type I error control. For window-based methods, controlling the false discovery rate over all detected windows does not guarantee control across all detected regions. Misinterpreting the former as the latter can result in unexpected liberalness. Here, several solutions are presented to maintain error control for these *de novo* counting strategies. For peak-based methods, peak calling should be performed on pooled libraries prior to the statistical analysis. For window-based methods, a hybrid approach using Simes’ method is proposed to maintain control of the false discovery rate across regions. More generally, the relative advantages of peak- and window-based strategies are explored using a range of simulated and real data sets. Implementations of both strategies also compare favourably to existing programs for differential binding analyses.

## INTRODUCTION

Chromatin immunoprecipitation with sequencing (ChIP-seq) is a widely used technique for the identification of protein binding sites in the genome. Traditionally, ChIP-seq experiments are analysed by comparing the ChIP library with a negative control (1) to detect regions of absolute enrichment. However, comparisons between ChIP-seq libraries can also be performed to identify changes in enrichment patterns between different biological conditions (2–4). Detection of differential binding (DB) between two conditions does not require a negative control library. This lowers costs when only relative binding is of interest. A DB analysis is also easier to interpret as DB regions are more likely to be relevant to the biological difference under investigation.

The first step in a DB analysis is to identify a set of genomic intervals over which DB is to be assessed. The number of sequence reads mapped within each interval is then counted. Each count serves as a measure of protein binding within that region. The simplest strategy is to concentrate on known genes and their putative promotor regions (3,5,6). For example, Pal *et al.* (3) counted the number of reads mapped to a fixed interval around the transcriptional start site of each gene. Each region was then tested for significant differences in the enrichment of epigenetic marks.

However, restricting the analysis to pre-defined regions is not always appropriate. Such an approach will obviously miss DB events at unexpected loci outside the pre-defined regions. It will also be unable to resolve DB events that vary within a pre-defined region. Consider a promoter region containing two transcription factor (TF) binding sites. Suppose that one site is bound by the target TF in one cell type, whereas the other site is bound with equal intensity in another cell type. When the two cell types are compared, a region-based method that counts reads across the entire promoter will yield identical counts for both cell types. This means that differences within the promoter will not be detected.

Hence, there is a need for DB strategies that identify the genomic regions of interest *de novo*. Two broad *de novo* strategies have been used in the literature. Peak-based methods count reads over observed peaks in read density (7,8) that have been identified using a peak-calling program, such as MACS (9). Window-based methods use a sliding window approach whereby the number of reads inside each window is counted (10). Both methods can detect DB events that might be missed by region-based counting. In the example above, a peak-based method will define each TF binding site as a distinct peak. Differences between cell types can then be detected for each site as each peak is analysed separately. A similar argument can be made for window-based approaches if each site is covered by a different window.

Once counts are obtained, DB loci are identified by testing for significant differences between conditions. The statistical analysis must be able to deal with low, overdispersed counts and a limited number of biological replicates. The software package edgeR (11) has previously been used for this purpose (2,3,12,13). After testing, the Benjamini–Hochberg (BH) method can be applied to control the false discovery rate (FDR) across detected loci (14). This corrects for multiple tests across the genome without being overly conservative. The detected DB features can then be correlated to differential gene expression or used to identify novel regulatory elements. The use of existing statistical procedures for the DB analysis is relatively straightforward, effective and (mostly) independent of the chosen read counting strategy.

A number of subtleties need to be considered to maintain statistical validity in a *de novo* DB analysis pipeline. For peak-based methods, a DB analysis on called peaks is performed with the same data set used to define said peaks. This means that peak-based methods use the same data twice (i.e. data snooping), which can result in loss of type I error control if not done correctly. For window-based methods, naïve application of the BH method provides control of the window-level FDR rather than the intended region-level FDR. Misinterpreting the former as the latter may result in loss of FDR control. Neither issue seems to have been appreciated in the implementation of existing methods for the *de novo* detection of DB regions.

This paper provides some recommendations to maintain error control for *de novo* counting strategies. For peak-based methods, peak calling should be performed on pooled libraries to avoid the loss of type I error control from data snooping. For window-based methods, region-level FDR control can be maintained by applying Simes’ method on *P*-values of clustered windows. Neither method is more powerful than the other for detection of all DB features. Rather, implementations of both strategies have complementary detection profiles on simulated and real data sets. Both implementations also outperform several existing programs for DB analyses.

## MATERIALS AND METHODS

### Estimation of the average fragment length

For each real ChIP-seq data set, the average fragment length was estimated using cross-correlation plots (15,16) as shown in Supplementary Figure S1. In each simulation, the average fragment length was set to the known true value of 100 bp.

### Implementation of the sliding window method

The sliding window method considers equispaced genomic windows and counts the number of bound DNA fragments overlapping each window. For TF data, each window was 1 bp in size to represent the narrowest possible feature. For histone mark data, a window width of 150 bp was used. This represents the length of DNA in a nucleosome and is the smallest relevant interval for histone mark enrichment (17). The spacing between successive window midpoints ranged from 25 to 100 bp. Larger spacings reduce computational work while smaller spacings provide greater resolution.

To represent the complete post-sonication DNA fragment from which the read was sequenced (18), each read was directionally extended to a length equal to the average fragment length. The number of fragments overlapping each window was then counted. This means that the count for each window will not only include reads within the window itself, but also any read lying immediately upstream of the window on the strand to which that read was mapped.

Finally, DB windows were identified by analysing the collected counts with edgeR. The BH method was applied to control the FDR across all detected windows.

### Implementation of the peak-based method

Reads from all libraries in a data set were pooled into a single library. MACS (v1.4.2) was then used in single-sample mode to call peaks from the pooled library (9). Model building was disabled and the shift size was manually set to half the average fragment length instead. Duplicate removal was also turned off. The genome size was set as the sum of the lengths of all chromosomes in the genome. Default values were used for all other MACS parameters. After peak calling, the number of 5′ read ends lying in the genomic interval corresponding to each peak was counted. This was repeated such that a count was obtained for each peak in each library. DB peaks were identified using edgeR and the BH method was applied to control the FDR across all detected peaks.

### Implementation of the hybrid approach

Reads were counted across windows as previously described. Peaks were also called using MACS as previously described. All windows overlapping at least one peak were identified and tested for DB with edgeR. Windows were then aggregated into peak clusters where each peak cluster contains all windows overlapping a corresponding peak. A combined *P*-value was computed for each peak cluster using Simes’ method (19). For a cluster containing *n* windows, the combined *P*-value is defined as

\begin{equation*} p_s = \min \lbrace np_r / r ; \; r = 1, 2 \dots , n\rbrace \end{equation*}

where the *p*_*r*_ are the individual window *P*-values sorted in increasing order. This provides weak control of the family-wise error rate across the set of null hypotheses for all windows in the cluster. In other words, *p*_*s*_ represents evidence against the global null hypothesis, i.e. that no windows in the cluster are DB. The BH method was then applied to the combined *P*-values to control the FDR across peak clusters. This is equivalent to region-level FDR control as each cluster represents a distinct genomic region.

### DB analysis with edgeR

Windows or peaks with low counts across all libraries were filtered out prior to the DB analysis. Low-abundance features are interpreted as ‘background’ regions of non-specific enrichment in which DB is not expected. Removal of these uninteresting features improves power by reducing multiple testing without compromising type I error control (20). For real data sets, windows or peaks were filtered out if the total count across all libraries was below 20 or if the average log_2_ count-per-million (as computed by the aveLogCPM function in the edgeR package) was less than −1. For simplicity, the aveLogCPM filter was omitted from analyses of simulated data for which library sizes are equal and non-specific enrichment is low or absent.

Scaling normalization is necessary to remove composition biases in sequencing data prior to comparisons between libraries (21). Here, a modified version of the trimmed mean of *M*-values (TMM) method (21) was used. Instead of applying the TMM method directly to the same read counts used for the DB analysis, a new table of read counts was generated for normalization purposes. For each library, reads were counted into contiguous 10 kbp bins across the genome based on the 5′ end location of each read. Normalization factors were estimated from these binned counts using the TMM method without any precision weighting. Large bins were used instead of windows to provide greater read counts for optimal normalizaton. The use of equisized bins also ensures that genomic regions are equally represented, e.g. regions with more called peaks do not contribute more *M*-values. Similarly, precision weights were not used to ensure that equal weight was assigned to all *M*-values. Further justification is given in the second section of Supplementary Materials and Supplementary Figure S2. This procedure assumes that most of the genome is not differentially bound by the protein of interest. Of those regions which are DB, no assumption is made regarding the direction of change. As implemented, the TMM method will tolerate up to 30% of bins that are DB in either direction, i.e. up to a maximum of 60% if the number of DB bins is equal in both directions. Also note that normalization was only performed for analyses of real data. All normalization factors were set to unity in the simulations as no composition biases were present.

Finally, counts for each window or peak were tested for DB using edgeR (v3.4.2). The edgeR package models the counts using negative binomial (NB) distributions. The estimateDisp function was used to estimate an abundance-dependent trend for the NB dispersions (22). To normalize for compositional biases, the effective library size for each sample was set as the product of the raw library size and the normalization factor described above. A quasi-likelihood (QL) *F*-test was then conducted for each peak or window using the glmQLFTest function (23), with robust estimation of the prior degrees of freedom (24).

### Outline of the simulation design

Data were simulated for a ChIP-seq experiment consisting of two biological replicates in each of two groups. Exactly *N* peaks separated by at least 10 kbp were added to an artificial genome containing a single chromosome. For each library in group *j*, the number of reads in peak *i* was sampled from a NB distribution with mean μ_*ij*_ and dispersion φ_*i*_. The latter was sampled from an inverse chi-squared distribution with 20 degrees of freedom. For each non-DB peak *i*, μ_*ij*_ was set to a constant *x*_0_ for all *j*.

DB was incorporated by picking *N*′ peaks and setting μ_*i*1_ = *x*_1_ and μ_*i*2_ = *x*_2_ for each chosen peak *i*, where *x*_1_ and *x*_2_ are non-equal constants. This was repeated with *N*′ different peaks after swapping *x*_1_ and *x*_2_. This means that each group is enriched for the same number of DB peaks. Thus, TMM normalization is not required as the balanced addition of DB peaks between groups avoids the introduction of any composition bias. All mean values were chosen such that *x*_1_ + *x*_2_ = 2*x*_0_ to ensure that no correlation was present between average abundance and DB.

Finally, the position and strand of each read were simulated. Reads were evenly distributed between strands. For TF simulations, the position of each forward- and reverse-mapping read was sampled from *c*_*i*_ − *fX* and *c*_*i*_ + *fX*, respectively, given the peak centre *c*_*i*_, the fragment length *f* = 100 and *X* = Beta(2, 2). This models the strand bimodality observed in real TF ChIP-seq data. For histone mark simulations, the position of each read was sampled from a scaled Beta(2, 2) distribution centred at *c*_*i*_. The scaling factor was set at 1000 bp to represent the diffuse nature of histone mark enrichment. Sampling is also strand-independent to reflect reduced strand bimodality for histone data.

The region defined by each peak is defined as the interval containing all reads associated with that peak. For TF simulations, this corresponds to [*c*_*i*_ − *f*, *c*_*i*_ + *f*] for each DB peak *i*. For histone simulations, the peak region is defined as [*c*_*i*_ − *w*/2, *c*_*i*_ + *w*/2] given the scaling factor *w* = 1000.

### Simulations to measure type I error control

A TF ChIP-seq data set was simulated with *x*_0_ = 20, *N* = 50 000 and no DB (i.e. *N*′ = 0). A number of peak-calling methods using MACS were applied to call peaks from the simulated data. Parameter settings for MACS were defined as previously described. The threshold on the consolidated *P*-value for each method was set such that 10 000 peaks were called. All called peaks were used if fewer than 10 000 were called. To avoid confusion, the known peaks in the simulation will be referred to as ‘true’ peaks, whereas those called from the simulated data will be referred to as ‘empirical’ peaks. For each method, all true peaks overlapping an empirical peak were identified. For each of these true peaks, the number of 5′ read ends lying in the corresponding genomic interval was counted in each library. Counts were then used in edgeR to compute a *P*-value for DB in that peak. The observed type I error rate was defined as the proportion of *P*-values below a specified threshold. As a reference, the counting and DB analysis were also repeated with 10 000 randomly sampled true peaks.

### Simulations to measure FDR control

Both TF and histone mark data sets were simulated with *x*_0_ = 50, *x*_1_ = 90, *x*_2_ = 10, *N* = 20 000 and *N*′ = 1500. The sliding window method was then used to analyse the simulated data. Windows were detected as differentially bound by controlling the FDR at 0.05. The observed window-level FDR was defined as the proportion of detected windows lying outside the regions corresponding to DB true peaks. The observed region-level FDR was defined as the proportion of detected true peaks (i.e. overlapped by at least one detected window) that were not DB true peaks. The hybrid approach was also applied to detect putative DB peak clusters at an FDR threshold of 0.05. In this case, the observed region-level FDR was defined as the proportion of detected true peaks (i.e. overlapping with a detected peak cluster) that were not DB true peaks.

### Simulations to measure relative performance

A simulated data set was generated as previously described with *N* = 20 000, *N*′ = 500 and *x*_0_ = 15, *x*_1_ = 25, *x*_2_ = 5 (for TF simulations) or *x*_0_ = 30, *x*_1_ = 50, *x*_2_ = 10 (for histone mark simulations). Non-specific enrichment was added by partitioning the genome into contiguous 2 kbp blocks. The number of reads in each block *k* was sampled from a NB(μ_*k*_, φ_*k*_) distribution. The mean μ_*k*_ was sampled from a *U*(10, 50) distribution to mimic uneven sequencing coverage in real data. The dispersion φ_*k*_ was sampled as previously described. Reads were evenly distributed between strands and uniformly distributed within each block. Note that μ_*k*_ and φ_*k*_ are constant between libraries. This ensures that regions of non-specific enrichment are not DB.

Several methods were then used to detect *de novo* DB regions from the simulated data using an FDR threshold of 0.05. The observed (region-level) FDR was defined as the proportion of regions detected by each method with no overlaps with the regions corresponding to DB true peaks. The detection power of each method was measured by counting the number of detected DB true peaks, i.e. DB true peaks that overlapped at least one detected region from that method.

### Processing of real data sets

Four ChIP-seq data series containing biological replicates in multiple groups were downloaded from the Gene Expression Omnibus (GEO) (25). Accession numbers are given in Table 3. Reads were aligned to the mm10 build of the mouse genome using Rsubread v1.10.5 (26) with a consensus threshold of 2. Alignments with mapping qualities below 100 were discarded. SAM files were compressed, sorted and indexed using SAMtools (27). Read processing statistics are described for each library in the first section of Supplementary Materials and Supplementary Table S1. During DB analyses, regions lying outside of autosomes (for STAT5) or nuclear chromosomes (others) were removed from the results before application of the BH method. This avoids detection of spurious differences due to copy number changes for loci on the sex chromosomes or the mitochondrial genome.

### Alternative software packages for DB analyses

Programs from the USeq suite (v8.6.8) were used to identify DB regions (28). Libraries were converted using the Tag2Point program and processed using the MultipleReplicaScanSeqs program with a peak shift of 50 bp and a window size of 100 or 250 bp for TF and histone mark data, respectively. Larger window sizes are necessary for a valid comparison to the settings used in the sliding window implementation. This is because reads are shifted by half the fragment length to account for bimodality, rather than being extended by the full fragment length. The EnrichedRegionMaker program was then used to identify DB regions at a specified FDR threshold. No filtering was performed on the log-fold change. Default values were used for all other parameters.

The diffReps pipeline (v1.55.4) was also used to perform a DB analysis (10). The fragment length was set to 100 bp and the window size was set at 100 or 250 bp for TF and histone mark data, respectively. Overlapping windows containing significant differential enrichment were selected and assembled into differential sites. DB regions were then defined as differential sites with adjusted *P*-values below a specified FDR threshold. Hotspot detection and genomic annotation were disabled. Otherwise, default parameter settings were used.

## RESULTS

### Outline of the DB problem

Consider a ChIP-seq experiment containing multiple replicate libraries for each of two biological conditions. The aim is to identify genomic regions with significant differences in protein binding between conditions. However, reads first need to be summarized into counts representing the strength of binding in each region. This is not trivial when the regions of interest are not known in advance. In such cases, peak- or window-based approaches must be used.

### Description of the peak-based strategy

Observed peaks in read density are identified from the data using programs like MACS. The number of reads lying within each identified peak is counted for each library. Counts can then be used to test for DB between conditions at the genomic position corresponding to the identified peak. However, some caution is required to avoid loss of type I error control when detecting DB peaks from the same data set used to define the peaks, i.e. data snooping.

### Many peak-based implementations are possible

A variety of approaches can be used to define peaks for a data set with multiple libraries and groups (Table 1). Peaks can be identified using MACS in single-sample mode for each individual library, or for each group after pooling all libraries in the group. Multiple peak sets across all libraries or groups can then be consolidated into a single list by intersection or union operations or some compromise thereof, e.g. requiring the peak to be present in at least two libraries. For example, Bardet *et al.* recommend taking the union of stringently called peaks from each condition (4). Peaks can be also identified by applying MACS in two-sample mode to the pooled libraries for each group, i.e. the pooled library for one group is treated as a control and used to detect relative differences in the pooled library for the other group. Alternatively, peaks can be called with MACS in single-sample mode after pooling reads across all libraries in the data set.

**Table 1.** Description of peak calling strategies. Each strategy is given an identifier and is described by the mode in which MACS is run, the libraries on which it is run and the consolidation operation (if any) performed to combine peaks between libraries or groups. For method 6, the union of the peaks in each direction of enrichment is taken.

ID	Mode	Library	Operation
1	Single-sample	Individual	Union
2	Single-sample	Individual	Intersection
3	Single-sample	Individual	At least 2
4	Single-sample	Pooled over group	Union
5	Single-sample	Pooled over group	Intersection
6	Two-sample	Pooled over group	Union
7	Single-sample	Pooled over all	–

The differences between these methods can be interpreted in terms of the peak-calling *P*-values produced by MACS. Let π_*lm*_ be the peak-calling *P*-value for peak *l* in an individual library *m*. The set of *P*-values across *ν* libraries for this peak can then be denoted as

\begin{equation*} P_l =\lbrace \pi _{l1}, \pi _{l2}, ..., \pi _{l\nu }\rbrace \end{equation*}

where π_*lm*_ = 1 if no peak corresponding to *l* was called in library *m*. A consolidation operation will summarize *P*_*l*_ into a single statistic, named here as the consolidated peak-calling *P*-value. A peak will only be retained after consolidation if its consolidated *P*-value is below some threshold value *τ*. For methods 1, 2 and 3, the consolidated *P*-value is defined as the minimum, maximum and second-smallest π_*lm*_ ∈ *P*_*l*_, respectively. For groups, π_*lm*_ can be redefined as the peak-calling *P*-value from the pooled library for each group *m*. This means that the consolidated *P*-value for methods 4 and 5 is the smallest and largest value in *P*_*l*_, respectively, where *P*_*l*_ is also redefined across groups. For methods 6 and 7, only one *P*-value is reported by MACS for each peak. This can then be directly used as the consolidated *P*-value.

Some care is required in choosing the value of τ for each method. The consolidated *P*-values from intersection-based methods will always be larger than those from union-based methods. Lower consolidated *P*-values will also be observed with pooled libraries where the read counts are higher. Thus, the use of a constant *τ* will result in differing total numbers of peaks from each method. This can confound a comparison between methods when DB is of interest. For example, a method that detects more DB peaks may only do so because the total number of called peaks is greater. One could overcome this by scaling the results such that the total number of peaks is the same for all methods. However, this will be misleading if the relationship between the total number of peaks and the number of DB peaks is non-linear. The safer approach is to set *τ* separately for each method such that the same number of peaks *C* is called by each method. This ensures that the results are directly comparable between methods. Note that *C* should be set low (i.e. stringent) enough so that most putative peaks are not called. Otherwise, the peak sets and DB results for each method will be identical.

The parametrization described above assumes that a one-to-zero-or-one relationship exists between the same peak in different libraries. The reality is more complicated due to slight differences in peak coordinates between libraries. In particular, the consolidation operations will have different effects on the final coordinates. Intersection operations will yield smaller peaks whereas union operations will yield larger peaks. This results in smaller and larger read counts, respectively, compounding the differences between methods. To simplify any comparison, the same peak intervals are used for read counting in each method. For example, the known genomic coordinates for each true peak in simulated data would be used instead of the empirically defined interval. This means that any differences between methods are not simply due to differences in peak widths after consolidation.

### Peaks should be called from pooled libraries

Each strategy in Table 1 was applied to simulated TF data to assess type I error control. For each simulated peak, the null hypothesis is true and the counts are exactly NB-distributed (as no additional reads were simulated to represent non-specific enrichment). This means that the *P*-values computed by edgeR should closely follow a uniform distribution. However, this is not observed for all methods (Table 2). The most dramatic loss of type I error control occurs in method 6. Here, all peaks have low *P*-values as they are defined by DB between groups. This results in an observed error rate well above the specified threshold. Loss of control is more subtle for method 4 where peaks with low average counts for each group are filtered out. Such peaks generally have larger *P*-values as the average count for each group is constrained to low values and, therefore, more likely to be similar between groups. Removal of these peaks results in the depletion of large *P*-values, a relative enrichment of small *P*-values and an increase in the proportional error.

**Table 2.** The observed type I error rate when testing for differential enrichment using counts from each peak calling strategy. Error rates for a range of specified error thresholds are shown. All values represent the mean of 10 simulation iterations with the standard error shown in brackets. RA: reference analysis using 10 000 randomly chosen true peaks.

ID	Error rate		
	0.01	0.05	0.1
RA	0.010 (0.000)	0.051 (0.001)	0.100 (0.002)
1	0.002 (0.000)	0.019 (0.001)	0.053 (0.001)
2	0.003 (0.000)	0.030 (0.000)	0.073 (0.001)
3	0.006 (0.000)	0.042 (0.001)	0.092 (0.001)
4	0.033 (0.001)	0.145 (0.001)	0.261 (0.002)
5	0.000 (0.000)	0.001 (0.000)	0.005 (0.000)
6	0.088 (0.006)	0.528 (0.013)	0.893 (0.006)
7	0.010 (0.000)	0.049 (0.001)	0.098 (0.001)

On the other hand, several methods are too conservative. For methods 1 and 3, peaks with consistently low counts across all individual libraries will not be called. These peaks generally have low variances as the counts are more likely to be similar between replicates. Their removal inflates the average variance which results in larger *P*-values upon application of empirical Bayes methods. In particular, the conservativeness of method 3 is understated here and should not be ignored (see third section of Supplementary Materials and Supplementary Figure S3). Intersection operations in methods 2 and 5 mean that the counts for all libraries or groups must be large for a successful peak call. This results in the depletion of small *P*-values as peaks with substantial differences between groups are ignored.

Loss of type I error control is obviously undesirable. Conservativeness is preferable but still problematic as it implies a concomitant drop in power. Indeed, the reference analysis in Table 2 suggests that it is possible to provide near-exact control in this simulation. The peak calling strategy that is closest to doing so is that using MACS on pooled libraries across the entire data set, i.e. method 7. For libraries of similar size, peak calling on pooled data effectively operates on the average abundance of each peak across all libraries. This is analogous to filtering on the overall mean in microarray data (20) and is approximately independent of the *P*-value from the DB analysis. That said, error control will not be maintained when pooling libraries with extreme size differences (see fourth section of Supplementary Materials and Supplementary Figure 4).

### Description of the window-based strategy

Window-based methods involve sliding a fixed-width window across the genome and counting the number of reads overlapping the window at each position. Each count serves as a measure of ChIP enrichment at the genomic interval corresponding to that window. For single-end data sets involving sharp regions of enrichment, reads can be directionally extended by the average fragment length (18). Each extended read represents the post-sonication fragment from which the read was sequenced. Counting can then be performed with the extended reads, rather than the original reads. This provides more accurate counts by accounting for strand bimodality. A similar approach involves shifting reads by half the average fragment length in the direction of the read to represent the midpoint of the putative fragment. The number of midpoints lying within each window can then be counted. Of course, read extension is not necessary for paired-end data as the boundaries of each fragment can be computed exactly, or for studying diffuse enrichment where the extension length is typically negligible relative to the size of the window.

### Window- and region-level FDRs are not equivalent

The output from peak- or region-based methods has an intuitive interpretation. The BH method can be applied to the *P*-values from each peak or pre-defined region to control the FDR, i.e. the proportion of detected peaks or regions that are false positives. Window-based approaches are more complicated as they generate results for overlapping windows. A naïve approach might be to apply the BH method to the *P*-values from all windows, in order to identify those containing significant differences. Overlapping DB windows can then be clustered into a single DB region to avoid redundancy in the results. However, the FDR being controlled here refers to the proportion of windows that are false positives, not the proportion of genomic regions represented by those windows. The latter is often more relevant as follow-up work is done on each region rather than each window position.

This difference in interpretation has practical consequences for the economy of validation. Consider an analysis where the FDR across all windows is controlled at 10%. In the DB results, there are 18 adjacent windows forming one cluster and 2 windows forming a separate cluster. Now, assume that the first cluster represents a true DB region whereas the second cluster is a false positive. A window-based interpretation of the FDR is correct as only 2 of the 20 window positions are false positives. However, validation will only be performed once on each region. This region-based interpretation results in an actual FDR of 50%, reducing validation efficiency and distorting the significance of putative DB events. Similar concerns have been raised in analyses of other types of spatial signals, such as neural imaging data (29,30).

**Figure 1.**
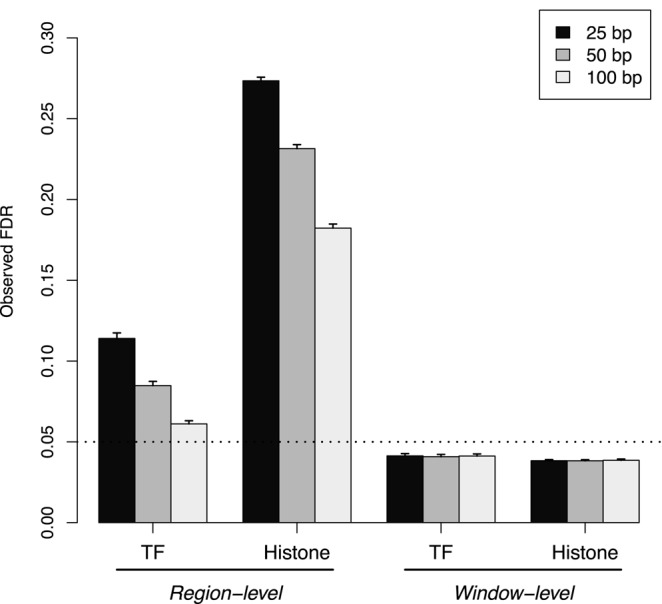
The observed region- and window-level FDR after applying the sliding window method to simulated TF and histone mark data. Spacing intervals ranging from 25 to 100 bp were tested. The FDR was nominally controlled at 0.05 (dotted line). Mean values are plotted along with the standard error across 10 simulation iterations.

The differences between the window- and region-level FDRs can be demonstrated by applying the sliding window approach to simulated TF and histone mark data. The sliding window method is able to control the window FDR below the specified threshold in both simulations (Figure 1). This is expected given that the BH method was applied to the set of window *P*-values. Note that *P*-values from adjacent or overlapping windows are highly correlated. The BH method is not guaranteed to maintain control in these conditions. However, it is known to be robust to correlations (31,32) which is consistent with the conservativeness in these results.

In contrast, control of the region-level FDR is lost at all spacing intervals. This is because all windows overlapping a strongly DB region will be detected (true positives), whereas only a few windows overlapping a non-DB region will be detected (false positives). When the results are interpreted in terms of regions, the total number of detected events will decrease as windows will not be counted separately. The imbalance in the distribution of detected windows between DB/non-DB regions means that the decrease in true positives will outweigh the decrease in false positives. This means that the observed FDR across regions will be greater than that across windows. Thus, control of the latter is insufficient to guarantee control of the former. Greater loss of control is also observed with lower spacings or histone marks. This is because the increase in the number of detected windows associated with each DB region inflates the difference between the window- and region-level FDRs.

### Region-level FDR can be controlled using Simes’ method

To avoid misinterpretation of the FDR, a hybrid approach is proposed whereby adjacent windows are clustered into ‘enriched regions’. The *P*-values for all windows in each region can then be combined using Simes’ method (19). The combined *P*-value represents the evidence against the global null hypothesis of each region, i.e. no window in the region is differentially bound by the protein of interest. Rejection of the global null indicates that the region is worth further examination as it contains one or more DB events. Application of the BH method to the set of combined *P*-values then controls the FDR across all detected regions.

Any clustering procedure can be used so long as it is blind to the DB status of the windows. Here, clusters are defined using the peaks called by MACS on pooled libraries, i.e. the set of all windows overlapping a peak is defined as a single enriched region. As previously shown, pooling ensures that the peak calls are independent of DB. Note that this hybrid approach is distinct from a peak-based method as the original *P*-values are still computed for each window. For small window sizes, this is equivalent to analysing read counts collected from each subsection of the peak. In comparison, a pure peak-based approach would compute *P*-values using counts collected across the entire peak.

**Figure 2.**
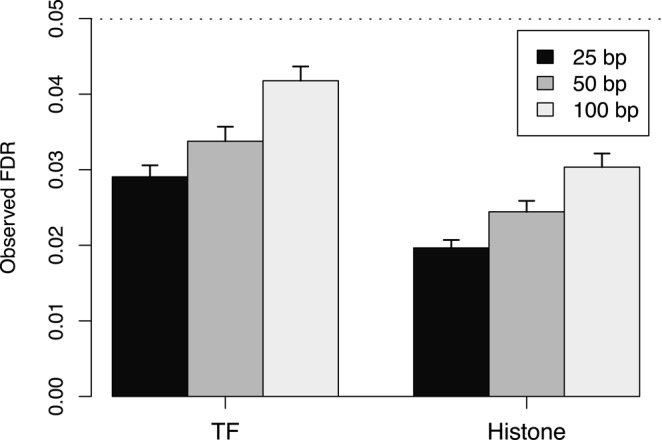
The observed region-level FDR at each spacing interval after applying the hybrid approach to simulated TF and histone mark data. The FDR was nominally controlled at 0.05 (dotted line). Mean values are plotted along with the standard error across 10 simulation iterations.

For testing, the hybrid approach was applied to simulated TF and histone mark data. At all examined spacing intervals, the hybrid approach controlled the region-level FDR below the specified threshold in both simulations (Figure 2). This corresponds to the appropriate rejection of the global null using the combined *P*-value for each enriched region. In addition, control is maintained despite the presence of strong correlations between overlapping windows. This is due to the robustness of Simes’ method to correlations (33,34). Indeed, Simes’ method is closely related to the BH method (14) which suggests that this robustness is common to both methods.

### Differences in FDRs are present in real data

The relevance of the simulation results was tested on several real data sets in Table 3. Both the hybrid approach and the sliding window method were used with a spacing of 50 bp to detect DB regions. For the latter, results were summarized by clustering DB windows less than 100 bp apart and with the same direction of fold change. Each cluster of windows represents a putative DB region. A non-negligible proportion of these clusters consist of more than one window (Supplementary Figure S6) which indicates that control of the window- and region-level FDRs will not be equivalent. Indeed, the set of DB regions detected with the sliding window method is almost a superset of that detected with the hybrid approach (Table 4). This is consistent with the liberalness of the former and represents the different consequences of controlling the region- and window-level FDRs in real data.

**Table 3.** Publicly available data sets used to compare DB detection power between methods. The GEO accession number, protein target and biological groups are shown for each data set. The number of biological replicates is also shown for each condition in brackets. All experiments were performed in mice. For GSE31578, one replicate was removed (*; see last section of Supplementary Materials and Supplementary Figure S5). FL, fetal liver-derived; DN, double negative cells.

Accession	Target	Groups	Ref.
GSE25533	NF-YA	Embryonic stem cells (2) and terminal neurons (2)	(35)
GSE31578	STAT5	Male liver (3) and female liver (2*)	(36)
GSE38046	H3K4me3	Pro-B cells (2) and mature B cells (2)	(37)
GSE31235	H3Ac	FLDN CD25^−^ cells (2) and thymus DN cells (2)	(38)

**Table 4.** Number of DB regions detected in each data set by the sliding window method or the hybrid approach at an FDR threshold of 0.05. The number of unique regions for each method is also shown. Regions were considered unique if there were no overlaps with detected regions from the other method. Note that counts may not be additive as regions shared between methods may not have a one-to-one correspondence.

	NF-YA	STAT5	H3K4me3	H3ac
Total sliding	2050	4261	33335	19099
Total hybrid	1366	1810	17961	4461
Sliding only	587	2240	7239	11762
Hybrid only	1	1	2	0

### Overview of existing software

A few existing programs are available for DB analyses of ChIP-seq data. diffReps is a sliding window method that performs read shifting to account for strand bimodality (10). After counting, DB windows are detected using an exact NB test. Differential sites are identified by aggregating DB windows and recomputing a *P*-value for each site. The BH method is then applied to control the FDR across all sites. While this controls the relevant FDR, a degree of data snooping is involved in the recomputation of the *P*-value for each site. USeq is another program that uses sliding windows with read shifting for count summarization (28). DB windows are detected using the DESeq package (39) with application of the BH method to control the FDR. Detected windows are then summarized into enriched regions. No consideration is given to the differences between the FDR across windows and that across enriched regions.

A near-infinite number of peak-based methods can be constructed by combining different peak calling programs with available statistical methods. In particular, several software packages can be used to facilitate the statistical analysis once peaks are obtained. The diffBind package merges a number of peak sets for a data set into a single consensus set (7). It then counts reads across the consensus peaks and performs a DB analysis using either edgeR or DESeq. Similarly, the DBChIP package consolidates multiple peak lists into a single set using hierarchical clustering (8). DB peaks are then identified by testing each consensus peak with edgeR or DESeq. Both packages provide a number of options for peak set consolidation. However, results from the earlier simulations suggest that only a few of these options will maintain type I error control during the DB analysis.

The performances of these existing methods were compared to that of the hybrid approach using simulated data. The aim is to determine which program provides the greatest detection power while maintaining FDR control. To increase the difficulty of DB detection, the intensities of all peaks in the simulation were reduced. Reads were also added in and around peaks to simulate non-specific enrichment. Increased difficulty ensures that the performances of different methods can be distinguished. To maintain type I error control (and for convenience), testing of the family of peak-based approaches was limited to a single method. Specifically, peaks were called by MACS on pooled libraries and tested for DB with edgeR. This is equivalent to method 7 in Table 1.

### Peak- and window-based methods favour detection of different features

**Figure 3.**
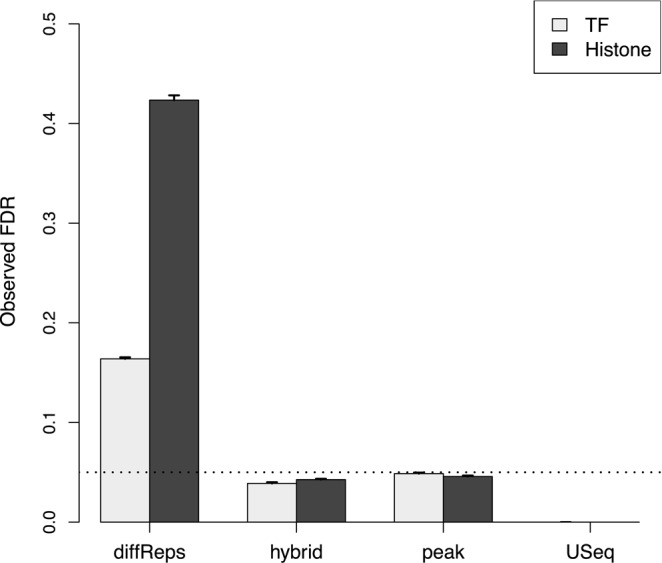
Estimates for the observed FDR after running each program on simulated TF and histone mark data with an FDR threshold of 0.05 (dotted). Values represent the mean of 10 simulations. Bars represent the standard error.

Both the peak-based method and the hybrid approach maintain the observed FDR below the specified threshold (Figure 3). This is consistent with the behaviour of the BH method when applied to *P*-values for peaks or regions. Of the other two programs, USeq is very conservative whereas diffReps is liberal. The performance of USeq is attributable to the conservativeness of DESeq compared to edgeR (40). For diffReps, loss of FDR control may be due to data snooping when DB windows are consolidated into ‘differential sites’. Recomputing a *P*-value for each site will have little meaning as the corresponding null hypothesis is false by definition.

**Figure 4.**
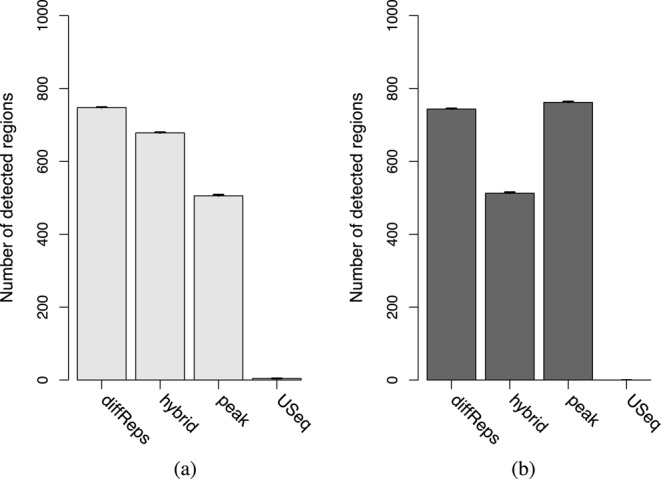
The number of detected peaks for each program in the simulated (a) TF and (b) histone mark data. All values represent the mean of 10 simulations. Bars represent the standard error.

For the TF simulations, the hybrid approach is more powerful than the peak-based approach (Figure 4a). This is because MACS cannot precisely estimate the boundaries of each peak. The median reported peak width is 494 bp whereas the true width is only 200 bp. This means that the count for each empirical peak in the peak-based method will include reads from non-DB background regions. Such ‘contamination’ weakens any DB fold change and reduces power. This is less of a concern for the hybrid approach as there is likely to be a window which overlaps the underlying true peak but not the surrounding background regions. The counts for this window will be (mostly) free of any contamination from background reads. This results in greater detection power for the underlying DB peak.

The situation is reversed for the histone mark simulation where the peak-based method is more powerful than the hybrid approach (Figure 4b). Here, the median reported width from MACS is 915 bp while the true width of each peak is 1000 bp. Counts can thus be obtained for the peak-based method without contamination from background regions. In comparison, each window in the hybrid approach is effectively 350 bp in width (150 bp with 100 bp directional extension). This means that the collected count for each empirical peak will larger than that for each window. As background contamination is not an issue, the difference in the size of the counts directly results in increased power for the peak-based method compared to the hybrid approach.

Of the other methods, diffReps detects the most peaks in the TF simulations and the second-most peaks in the histone mark simulations. This does not represent superior detection power as FDR control is not maintained. At the other extreme, USeq detects almost no peaks due to its conservativeness. This suggests that the peak-based method and the hybrid approach are valid alternatives to existing pipelines as both provide substantial detection power while maintaining FDR control.

### Different detection preferences are preserved in real data

The simulations indicate that the hybrid approach and the peak-based method favour detection of different ChIP targets. This was tested by applying each method to real data. Both methods detected a comparable number of regions across all data sets (Table 5) with no consistent difference between the TF and histone mark experiments. However, closer examination revealed that each method detected a substantial number of unique regions in each data set. This suggests that different types of DB features were identified by each method, consistent with the simulation results.

**Table 5.** Number of DB regions detected in each data set by the hybrid approach and the peak-based method. The number of regions unique to each method is also shown.

	NF-YA	STAT5	H3K4me3	H3ac
Total peak	875	1949	21365	2238
Total hybrid	1366	1810	17961	4461
Peak only	286	844	4000	615
Hybrid only	777	705	596	2838

These detection preferences can be illustrated by examining the DB regions unique to each method. Peak-based methods favour detection of diffuse DB regions where greater counts can be collected over a larger region (Supplementary Figure S7a). In contrast, window-based methods can distinguish between a DB region and the neighbouring non-DB background with greater ease than peak-based methods. Specifically, at least one window can overlap and represent the underlying true peak without being contaminated by reads from the surrounding background regions. This allows the detection of DB events which would otherwise be missed when peak calling is not precise (Supplementary Figure S7b). Both of these examples are consistent with the behaviour observed in the simulated data.

Complex scenarios can also be considered where a peak shifts (e.g. nucleosome sliding) or shrinks (e.g. chromatin spreading) between conditions. Both events will manifest as DB within a larger enriched region (Supplementary Figure S8). For window-based strategies, each subinterval of the enriched region corresponds to a separate window. This means that DB subintervals in shifted or shrunken peaks can be easily identified (Supplementary Figure S7c). In contrast, the peak-based method will define the entire enriched region as a single empirical peak. This is because no distinction can be made between shrunken/shifted peaks after pooling. Thus, detection of DB subintervals is likely to be suboptimal as the counts for each empirical peak are collected across the entire region.

The conclusions presented here can also be generally applied to diffReps and USeq. Both are window-based methods which should provide some robustness when analysing complex DB events. Neither method is directly tested as the use of a different statistical framework has a substantial impact on the total number of detected features. This confounds the comparison between methods by masking the contribution of different read counting strategies. More generally, caution is required when interpreting results for real data where the true/false positives among the putative DB regions are unknown. This precludes any conclusions with respect to the relative power or specificity of each method.

## DISCUSSION

Some consideration of error rates is necessary when peak- or window-based read counting is performed for a DB analysis. For peak-based methods, reads should be pooled across the data set prior to peak calling. This avoids the loss of type I error control typically associated with data snooping. For window-based methods, control of the FDR across windows does not guarantee control of the FDR across genomic regions. This has practical consequences as regions are more relevant than individual windows. Loss of control of the region-level FDR distorts the significance of DB events which may lead to incorrect biological conclusions. It also reduces the efficiency of follow-up work as the proportion of false positives can be substantially higher than the specified value.

Here, a hybrid approach is presented which uses Simes’ method to combine *P*-values across all windows in a peak. This restores control of the region-level FDR while retaining the robustness of window-based methods. The hybrid approach is more reliable than a peak-based method for complex DB events involving peak shifting, shrinkage or non-negligible background enrichment. On the other hand, peak-based methods are more powerful for detecting diffuse DB regions. This complementarity is observed in both real and simulated data for TF and histone mark ChIP-seq experiments. Both of the implementations tested here also compare favourably to existing pipelines for window-based DB analyses when tested on simulated data.

Of course, only a small number of possible implementations have been tested in this paper. Different peak calling programs or parameters can be used to obtain smaller intervals for the peak-based methods (41) to improve resolution of sharp features. Conversely, the use of larger window sizes in the hybrid approach can favour detection of diffuse DB regions. The behaviour of these alternative implementations can generally be predicted from the size of the intervals used for read counting. All methods assume that DB occurs in the same direction throughout the interval, i.e. the DB status is constant. Increasing the interval size will strengthen this assumption and increase counts at the cost of spatial resolution. At the other extreme, care may be required to avoid misinterpretation of the FDR if multiple small peaks are called for each DB event.

So, which approach should be used for routine analyses of ChIP-seq data? Neither class of methods is consistently more powerful than the other. The family of window-based methods has a more conservative philosophy as fewer assumptions are made on the nature of DB features. In particular, the assumption of a constant DB status throughout each window is weakened by the use of small windows. This suggests that window-based methods are safer to use as any strongly DB region will be detected, regardless of the circumstances. Windows can also be tiled across promoters or genes to improve spatial resolution in analyses using pre-defined regions. In contrast, peak-based methods use more aggressive assumptions when defining peaks and counting reads. This may be useful in squeezing more information from data sets with low counts or subtle changes.

This paper focuses on how genomic intervals are chosen for a DB analysis, and how these choices contribute to error rate control. In principle, the conclusions presented here should be independent of the choice of alignment algorithm, statistical test or normalization method. In the real data analyses, we mapped reads using subread, normalized using the TMM method with equispaced windows and conducted statistical tests using QL *F*-tests. These methods give good results in our hands, but the qualitative conclusions presented above should remain if other appropriate methods were substituted. The simulation comparisons on the paper were conducted in such a way that neither alignment or normalization was required.

All methods presented in this paper were written in the R or C/C++ programming languages, with extensive use of code from the Bioconductor project (42). Simulation scripts were also implemented in R and are available on request.

## SUPPLEMENTARY DATA

Supplementary Data are available at NAR Online.

SUPPLEMENTARY DATA

## References

[1] Liu E.T., Pott S., Huss M. (2010). Q&A: ChIP-seq technologies and the study of gene regulation. BMC Biol..

[2] Ross-Innes C.S., Stark R., Teschendorff A.E., Holmes K.A., Ali H.R., Dunning M.J., Brown G.D., Gojis O., Ellis I.O., Green A.R. (2012). Differential oestrogen receptor binding is associated with clinical outcome in breast cancer. Nature.

[3] Pal B., Bouras T., Shi W., Vaillant F., Sheridan J.M., Fu N., Breslin K., Jiang K., Ritchie M.E., Young M. (2013). Global changes in the mammary epigenome are induced by hormonal cues and coordinated by Ezh2. Cell Rep..

[4] Bardet A.F., He Q., Zeitlinger J., Stark A. (2011). A computational pipeline for comparative ChIP-seq analyses. Nat. Protoc..

[5] Young M.D., Willson T.A., Wakefield M.J., Trounson E., Hilton D.J., Blewitt M.E., Oshlack A., Majewski I.J. (2011). ChIP-seq analysis reveals distinct H3K27me3 profiles that correlate with transcriptional activity. Nucleic Acids Res..

[6] Statham A.L., Strbenac D., Coolen M.W., Stirzaker C., Clark S.J., Robinson M.D. (2010). Repitools: an R package for the analysis of enrichment-based epigenomic data. Bioinformatics.

[7] Stark R., Brown G. (2011). DiffBind: Differential Binding Analysis of ChIP-Seq Peak Data.

[8] Liang K., Keleş S. (2012). Detecting differential binding of transcription factors with ChIP-seq. Bioinformatics.

[9] Zhang Y., Liu T., Meyer C.A., Eeckhoute J., Johnson D.S., Bernstein B.E., Nusbaum C., Myers R.M., Brown M., Li W. (2008). Model-based analysis of ChIP-Seq (MACS). Genome Biol..

[10] Shen L., Shao N.Y., Liu X., Maze I., Feng J., Nestler E.J. (2013). diffReps: detecting differential chromatin modification sites from ChIP-seq data with biological replicates. PLoS ONE.

[11] Robinson M.D., McCarthy D.J., Smyth G.K. (2010). edgeR: a Bioconductor package for differential expression analysis of digital gene expression data. Bioinformatics.

[12] Chandra T., Kirschner K., Thuret J.Y., Pope B.D., Ryba T., Newman S., Ahmed K., Samarajiwa S.A., Salama R., Carroll T. (2012). Independence of repressive histone marks and chromatin compaction during senescent heterochromatic layer formation. Mol. Cell.

[13] Ward M.C., Wilson M.D., Barbosa-Morais N.L., Schmidt D., Stark R., Pan Q., Schwalie P.C., Menon S., Lukk M., Watt S. (2013). Latent regulatory potential of human-specific repetitive elements. Mol. Cell.

[14] Benjamini Y., Hochberg Y. (1995). Controlling the false discovery rate: a practical and powerful approach to multiple testing. J. Royal Stat. Soc. B.

[15] Kharchenko P.V., Tolstorukov M.Y., Park P.J. (2008). Design and analysis of ChIP-seq experiments for DNA-binding proteins. Nat. Biotechnol..

[16] Landt S.G., Marinov G.K., Kundaje A., Kheradpour P., Pauli F., Batzoglou S., Bernstein B.E., Bickel P., Brown J.B., Cayting P. (2012). ChIP-seq guidelines and practices of the ENCODE and modENCODE consortia. Genome Res..

[17] Humburg P., Helliwell C.A., Bulger D., Stone G. (2011). ChIPseqR: analysis of ChIP-seq experiments. BMC Bioinformat..

[18] Robertson G., Hirst M., Bainbridge M., Bilenky M., Zhao Y., Zeng T., Euskirchen G., Bernier B., Varhol R., Delaney A. (2007). Genome-wide profiles of STAT1 DNA association using chromatin immunoprecipitation and massively parallel sequencing. Nat. Methods.

[19] Simes R.J. (1986). An improved Bonferroni procedure for multiple tests of significance. Biometrika.

[20] Bourgon R., Gentleman R., Huber W. (2010). Independent filtering increases detection power for high-throughput experiments. Proc. Natl. Acad. Sci. U.S.A..

[21] Robinson M.D., Oshlack A. (2010). A scaling normalization method for differential expression analysis of RNA-seq data. Genome Biol..

[22] McCarthy D.J., Chen Y., Smyth G.K. (2012). Differential expression analysis of multifactor RNA-Seq experiments with respect to biological variation. Nucleic Acids Res..

[23] Lund S.P., Nettleton D., McCarthy D.J., Smyth G.K. (2012). Detecting differential expression in RNA-sequence data using quasi-likelihood with shrunken dispersion estimates. Stat. Appl. Genet. Mol. Biol..

[24] Phipson B., Lee S., Majewski I.J., Alexander W.S., Smyth G.K. (2013). Empirical Bayes in the presence of exceptional cases, with application to microarray data.

[25] Barrett T., Troup D.B., Wilhite S.E., Ledoux P., Rudnev D., Evangelista C., Kim I.F., Soboleva A., Tomashevsky M., Marshall K.A. (2009). NCBI GEO: archive for high-throughput functional genomic data. Nucleic Acids Res..

[26] Liao Y., Smyth G.K., Shi W. (2013). The Subread aligner: fast, accurate and scalable read mapping by seed-and-vote. Nucleic Acids Res..

[27] Li H., Handsaker B., Wysoker A., Fennell T., Ruan J., Homer N., Marth G., Abecasis G., Durbin R., 1000 Genome Project Data Processing Subgroup (2009). The Sequence Alignment/Map format and SAMtools. Bioinformatics.

[28] Nix D.A., Courdy S.J., Boucher K.M. (2008). Empirical methods for controlling false positives and estimating confidence in ChIP-Seq peaks. BMC Bioinformat..

[29] Benjamini Y., Heller R. (2007). False discovery rates for spatial signals. J. Am. Stat. Assoc..

[30] Chumbley J.R., Friston K.J. (2009). False discovery rate revisited: FDR and topological inference using Gaussian random fields. Neuroimage.

[31] Reiner A., Yekutieli D., Benjamini Y. (2003). Identifying differentially expressed genes using false discovery rate controlling procedures. Bioinformatics.

[32] Kim K.I., van de Wiel M.A. (2008). Effects of dependence in high-dimensional multiple testing problems. BMC Bioinformat..

[33] Samuel-Cahn E. (1996). Is the Simes improved Bonferroni procedure conservative?. Biometrika.

[34] Sarkar S.K., Chang C.K. (1997). The Simes method for multiple hypothesis testing with positively dependent test statistics. J. Am. Stat. Assoc..

[35] Tiwari V.K., Stadler M.B., Wirbelauer C., Paro R., Schübeler D., Beisel C. (2012). A chromatin-modifying function of JNK during stem cell differentiation. Nat. Genet..

[36] Zhang Y., Laz E.V., Waxman D.J. (2012). Dynamic, sex-differential STAT5 and BCL6 binding to sex-biased, growth hormone-regulated genes in adult mouse liver. Mol. Cell. Biol..

[37] Revilla-I-Domingo R., Bilic I., Vilagos B., Tagoh H., Ebert A., Tamir I.M., Smeenk L., Trupke J., Sommer A., Jaritz M. (2012). The B-cell identity factor Pax5 regulates distinct transcriptional programmes in early and late B lymphopoiesis. EMBO J..

[38] Zhang J.A., Mortazavi A., Williams B.A., Wold B.J., Rothenberg E.V. (2012). Dynamic transformations of genome-wide epigenetic marking and transcriptional control establish T cell identity. Cell.

[39] Anders S., Huber W. (2010). Differential expression analysis for sequence count data. Genome Biol..

[40] Law C.W., Chen Y., Shi W., Smyth G.K. (2014). Voom: precision weights unlock linear model analysis tools for RNA-seq read counts. Genome Biol..

[41] Laajala T.D., Raghav S., Tuomela S., Lahesmaa R., Aittokallio T., Elo L.L. (2009). A practical comparison of methods for detecting transcription factor binding sites in ChIP-seq experiments. BMC Genom..

[42] Gentleman R.C., Carey V.J., Bates D.M., Bolstad B., Dettling M., Dudoit S., Ellis B., Gautier L., Ge Y., Gentry J. (2004). Bioconductor: open software development for computational biology and bioinformatics. Genome Biol..

