# Supplementary material for: De novo detection of differentially bound regions for ChIP-seq data using peaks and windows: controlling error rates correctly
Source: Nucleic Acids Res. 2014 May 22;42(11):e95. doi: 10.1093/nar/gku351 (PMC4066778; doi:10.1093/nar/gku351)
Supplement: SUPPLEMENTARY DATA [file supp_42_11_e95__index.html]

 De novo detection of differentially bound regions for ChIP-seq data using peaks and windows: controlling error rates correctly — SUPPLEMENTARY DATA 

# *De novo* detection of differentially bound regions for ChIP-seq data using peaks and windows: controlling error rates correctly

## SUPPLEMENTARY DATA

**Files in this Data Supplement:**

- SUPPLEMENTARY DATA
