# Supplementary material for: De novo detection of differentially bound regions for ChIP-seq data using peaks and windows: controlling error rates correctly
Source: Nucleic Acids Res. 2014 May 22;42(11):e95. doi: 10.1093/nar/gku351 (PMC4066778; doi:10.1093/nar/gku351)
Supplement: SUPPLEMENTARY DATA [file supp_gku351_nar-00361-met-k-2014-File001.pdf]

Supplementary Materials For

De novo detection of differentially bound regions for ChIP-seq data using peaks and windows: controlling error rates correctly

by

Aaron T. L. Lun[1,2] and Gordon K. Smyth[1,3]

[1]The Walter and Eliza Hall Institute of Medical Research, 1G Royal Parade, Parkville, VIC 3052, Australia

[2]Department of Medical Biology, The University of Melbourne, Parkville, VIC 3010, Australia

[3]Department of Mathematics and Statistics, The University of Melbourne, Parkville, VIC 3010, Australia

8 March 2014

Details of read processing

Mapping diagnostics for each library are shown in Table S1. Each library contained 4 - 40 million reads ranging from 35 to 38 bp in length. The proportion of reads successfully mapped in each library ranged from 70 to 90%. Up to 50% of mapped reads were marked as potential PCR duplicates with the MarkDuplicates tool from the Picard suite (`http://picard.sourceforge.net`). Up to 39% of mapped reads had mapping quality (MAPQ) scores less than 100. Any technical replicates (i.e., multiple sequencing runs of the same library) were pooled into a single library prior to analysis. Final library sizes ranged from 7 million to 30 million reads.

For each dataset, reads with high MAPQ scores were pooled into a single library and used to construct cross-correlation plots [15]. The average fragment length was defined at the delay distance with the largest correlation value in the plot (Figure S1). This exploits the strand bimodality observed for narrow regions of enrichment. The spike at the read length is an artifact [16] and can be ignored. The plots also suggest that strand bimodality is present for the tested histone marks. This is consistent with the tightly localised nature of both marks.

Note that the cross-correlation plots were constructed after removing marked duplicates in each library. This improves the visibility of the fragment length peak by reducing the size of the spike at the read length. However, it must be stressed that marked reads were not removed in any of the DB analyses. Duplicate removal effectively caps the read density in each region. Detection power is subsequently reduced for high-abundance regions as all libraries have the same capped count. Alternatively, this can result in false positives for a DB comparison when the same upper bound is applied to libraries of differing sizes.

Justification for the normalization strategy

Binning is used to increase the size of the counts before TMM normalization. This means that an arbitrary prior value does not need to be added to avoid undefined M-values from zero counts. The precision of the M-values is also higher for larger counts [21]. This increases the effectiveness of trimming as the distinction between DB and non-DB bins is clearer. That said, bins should still be small enough so that DB and non-DB regions are separated. The choice of bin size depends on the library size as well as the degree of composition bias. An appropriate value will yield in a single mass of points corresponding to non-DB background regions in an MA plot (Figure S2). Multiple discrete masses indicate that the counts per bin are too low.

By default, the TMM method places more weight on M-values derived from larger counts when computing the normalization factor [21]. Precision weights are calculated by modelling the underlying counts with a Poisson distribution. This means that the contribution of enriched regions with large counts is much greater than that of background regions with small counts. However, differential binding is more likely to occur in the former. If any DB regions survive trimming (e.g. those with subtle fold changes), upweighting them will be counterproductive. To avoid this problem, equal weights are placed on the M-values from all bins remaining after trimming.

Understated conservativeness in peak calling

After pooling, peak calling with method 3 is the closest to providing exact type I error control. Here, the performances of these two methods are explored in more detail. NB-distributed counts were simulated for 500000 peaks with a mean of 20 and a dispersion of 0.05. This was done for an experiment with 2 replicates in each of 2 groups. To mimic method 7, rows were filtered to remove rows with count sums below a threshold. This is because pooling is equivalent to operating on the sum of counts for each peak.

Alternatively, to mimic method 3, only rows with at least two counts above a threshold were retained. In both cases, thresholds were chosen such that only 20% of rows were kept. Significant differences between groups were then identified using edgeR as previously described.

The ratio between the observed and specified type I error rates was examined for a range of error thresholds. Row sum filtering holds its size as the observed and specified error rates are similar for all tested thresholds, i.e., the ratio is close to 1 (Figure S3). Filtering by two or more libraries (method 3) is conservative as the specified error is much higher than the observed error. So far, this is consistent with previous simulation results. However, the conservativeness of method 3 is substantially more pronounced at lower p-values. This is important as small p-values are arguably the most important in practical settings. Severe multiple testing corrections in large datasets render large p-values irrelevant when searching for significant differences.

In summary, the near-expected error rates at thresholds of 0.05 and 0.1 in Table 2 are flattering to method 3 given its deteriorating performance at lower thresholds. This justifies the recommendation of library pooling prior to peak calling (method 7) as well as the more general use of row sum filtering or equivalents for NB-distributed count data. Note that the examination of lower thresholds is not feasible in Table 2 as there are insufficient peaks in the simulation for reliable estimation of error rates. Increasing the number of peaks results in a non-trivial increase in computational work as individual reads must be simulated and analyzed for each peak.

Liberalness in pooling with extreme library sizes

As previously mentioned, pooling is equivalent to operating on the sum of counts for each peak. The row sum is trivially transformed to the overall mean under a NB model when library sizes are equal. By analogy to the normal case [20], the overall mean (and thus, the row sum) will be independent of the DB status and the dispersion estimate for that peak. However, independence is lost when library sizes are very different. This is because the overall NB mean is no longer simply the sum of counts for each peak. Rather, it must be computed numerically using GLM-based methods such as those in edgeR [22].

The loss of independence can be demonstrated with a simple simulation. A matrix of NB-distributed counts was generated for 100000 peaks in an experiment with 2 replicates in each of 2 groups. The mean was set to 500 for all libraries in one group and 10 for all libraries in the other group. The dispersion was fixed at 0.05. Filtering was then performed to remove 80% of the lowest row sums. Alternatively, the same proportion of rows with the lowest overall NB mean was removed. Significant differences between groups were then identified using edgeR as previously described. Examination of the p-values indicates that row sum filtering loses control of type I error whereas filtering on the overall NB mean does not (Figure S4). This is consistent with the non-equivalence between the overall NB mean and the row sum when library sizes are substantially different.

Replicate removal for GSE31578

Library SRR340063 was removed from the STAT5 dataset prior to analysis. This library is an outlier in a multi-dimensional scaling plot (Figure S5) which indicates that it is substantially different from replicates in the same group. Including SRR340063 in the analysis increased the estimated common NB dispersion from 0.21 to 0.49 and the median estimated prior degrees of freedom from 21.7 to infinity. Larger dispersions with reduced variability are consistent with a confounding batch effect. The normalization factor for SRR340063 is approximately 0.64 whereas the factors for all other libraries are greater than 1. This indicates that it has composition bias - and thus, differential binding - relative to all other libraries, including the two replicates in the same group.

Table S1: Read processing diagnostics for datasets in Table 3. The protein target, biological condition, replicate number, Sequence Read Archive accession number, total number of reads, number of successfully mapped reads, number of unmarked reads and number of reads retained after filtering on MAPQ $\geq$ 100 are shown. Libraries with the same replicate number represent technical replicates (i.e., repeated sequencing runs). ESC: embryonic stem cells, TN: terminal neurons, FL: fetal liver-derived, DN: double negative.

Target	Condition	Rep	Accession	Total	Mapped	Unmarked	Retained
NF-YA	ESC	1	SRR074398	32038452	26336045	20226895	20003377
		2	SRR074399	36749276	30029025	19758793	23400935
	TN	1	SRR074417	39283051	32080626	22324725	26645252
		2	SRR074418	35423633	29886263	25000372	25018997
STAT5	Male	1	SRR340058	22986178	20874011	10171250	17571586
		2	SRR340059	17534466	15889306	12441784	12936732
		3	SRR340060	16958711	15273178	11633833	12675118
	Female	1	SRR340061	9970569	8854822	7673000	6890815
		2	SRR340062	8449482	7326103	5992202	5229646
		3	SRR340063	7897736	7035866	5116278	5627878
H3K4me3	pro-B	1	SRR499714	6186290	5626176	4944706	4427344
		1	SRR499715	6287942	5706448	5002078	4450511
		2	SRR499716	5119582	4616660	4151529	3740449
		2	SRR499717	5108112	4602512	4137226	3723158
	mature B	1	SRR499729	4187277	3700136	2985190	2646494
		1	SRR499730	5474498	4763670	3888904	3303306
		1	SRR499731	4668997	4083438	3275757	2827836
		2	SRR499732	4738253	4022416	3191926	2487979
		2	SRR499733	4587785	3876875	3060460	2357655
H3ac	FLDN cells	1	SRR330784	9853120	7800569	7364519	6140419
		1	SRR330785	15719521	12444530	11665007	9783929
		2	SRR330786	22732516	17098728	16022911	12684711
	Thymus DN	1	SRR330792	13863504	9098161	8531302	6509858
		1	SRR330793	12532671	8213842	7722051	5887280
		2	SRR330794	26209223	21490404	19926078	18542654

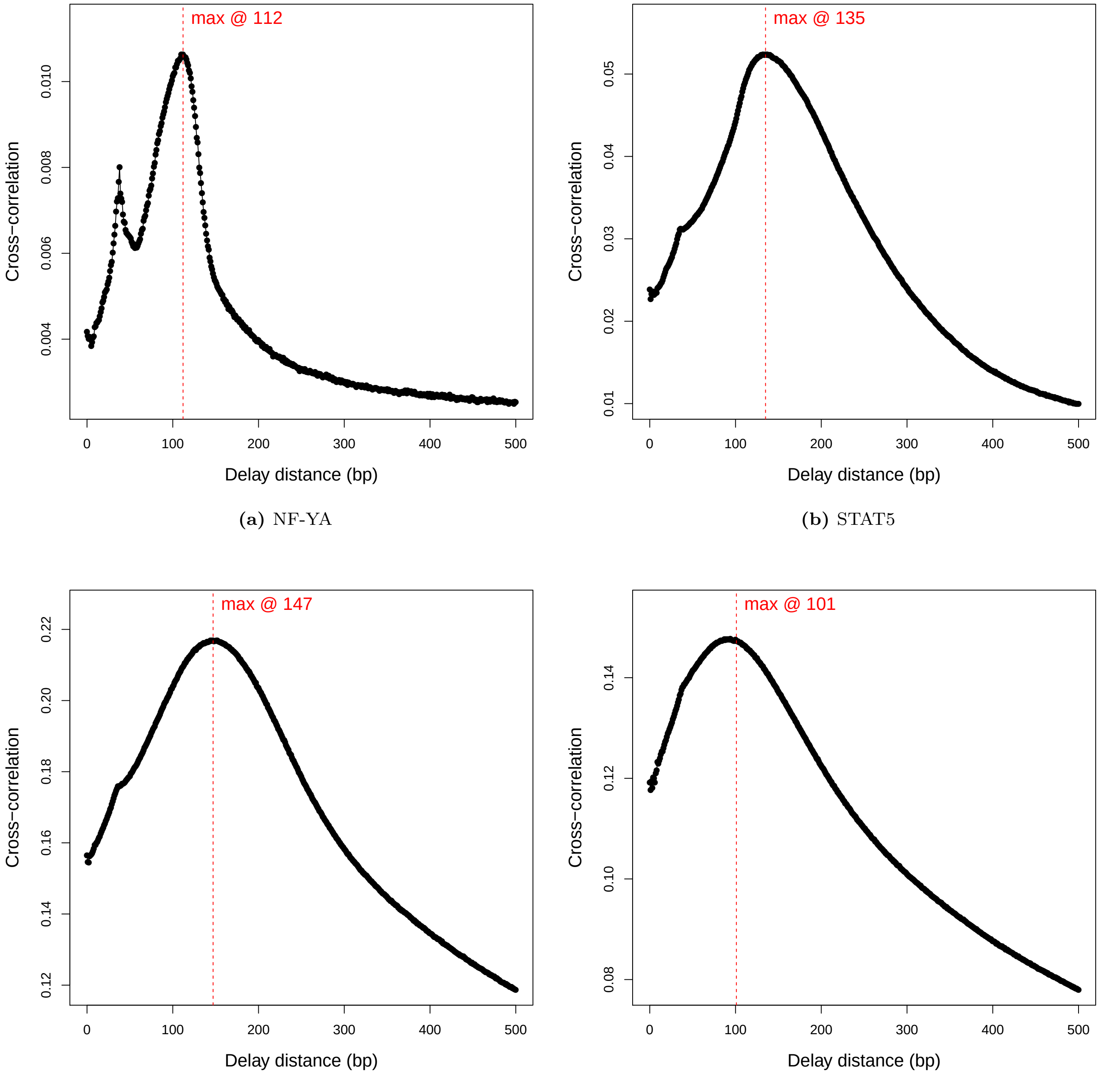

(a) NF-YA

(b) STAT5

(c) H3K4me3

(d) H3ac

Figure S1: Cross-correlation plots of pooled libraries for all datasets. The red line marks the delay distance with the maximum correlation value, after ignoring the spike at the read length.

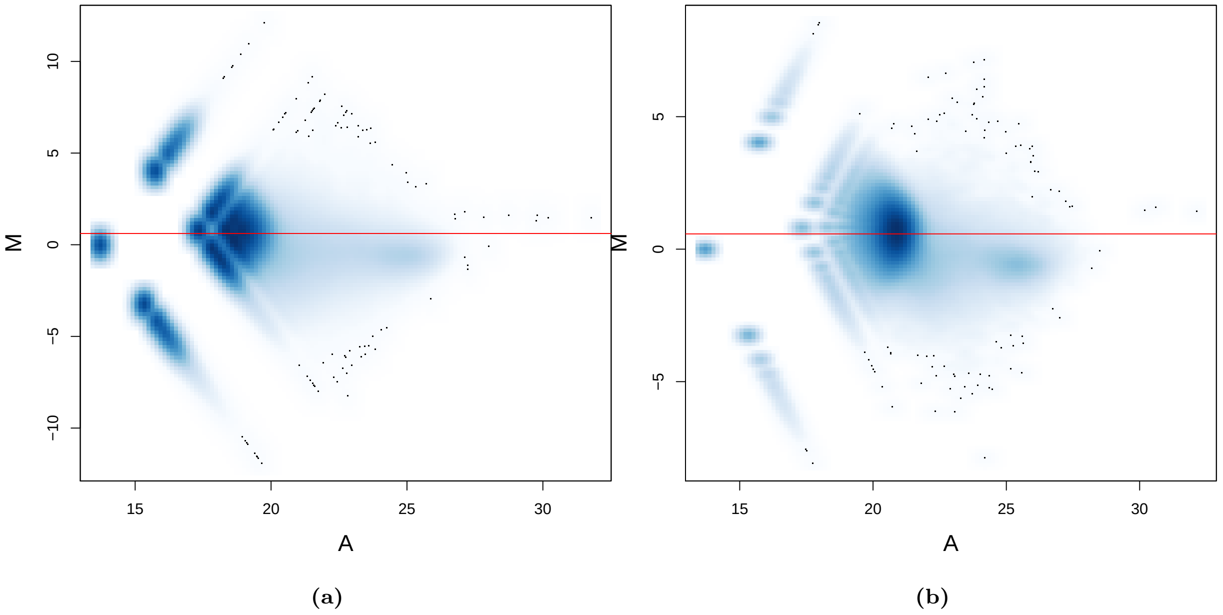

Figure S2: MA plots for the H3K4me3 dataset between pro-B and mature B libraries using counts from (a) 2 and (b) 10 kbp bins. The depth of colour at any point in the plot corresponds to the number of bins at those coordinates. The red line represents the M-value corresponding to the estimated normalization factor.

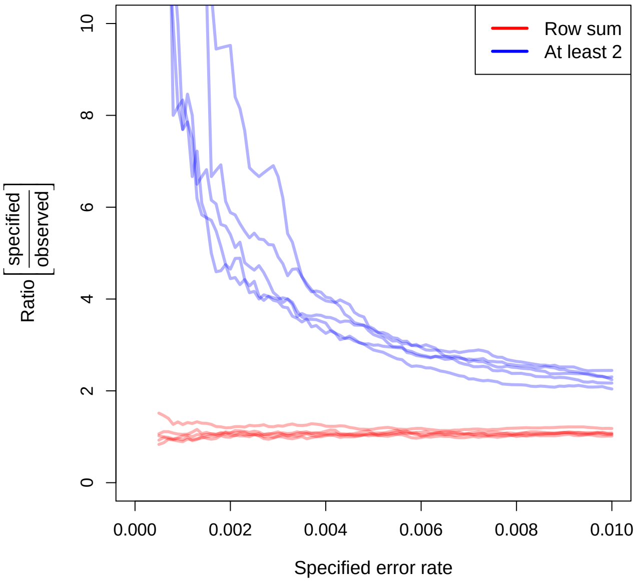

Figure S3: Comparison of the observed type I error rates with the specified thresholds for both filtering strategies. Each line represents the results from a single simulation run (5 runs in total).

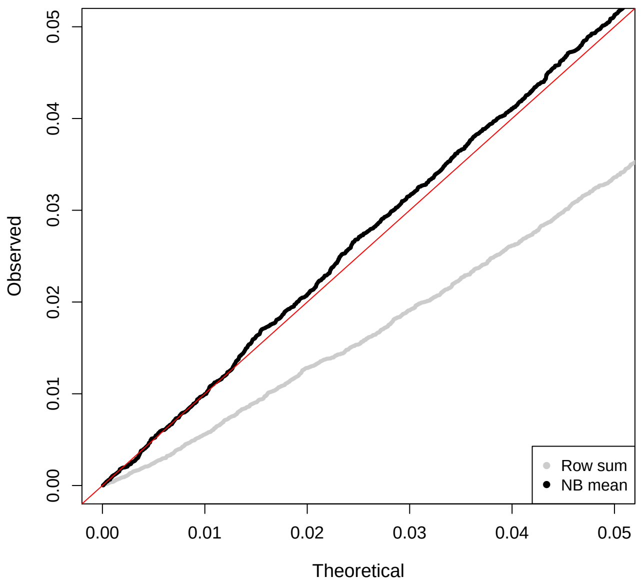

Figure S4: Comparison of the observed p-values with the theoretical quantiles for a uniform distribution after filtering on the row sum and the overall NB mean. The red line represents the expected curve under the null.

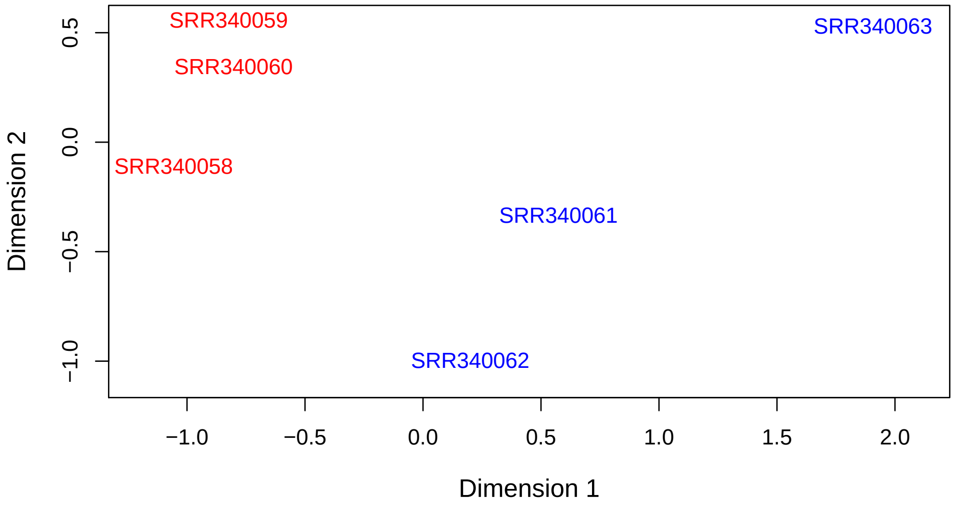

Figure S5: Multidimensional scaling plot for all replicate libraries in the STAT5 dataset. Distances were calculated by counting reads into contiguous 1000 bp bins, identifying the top 1000 most variable bins and calculating the square root of the average squared log-fold change between each pair of libraries across those bins. Each library is labelled with its Sequence Read Archive accession for male (red) and female (blue) samples.

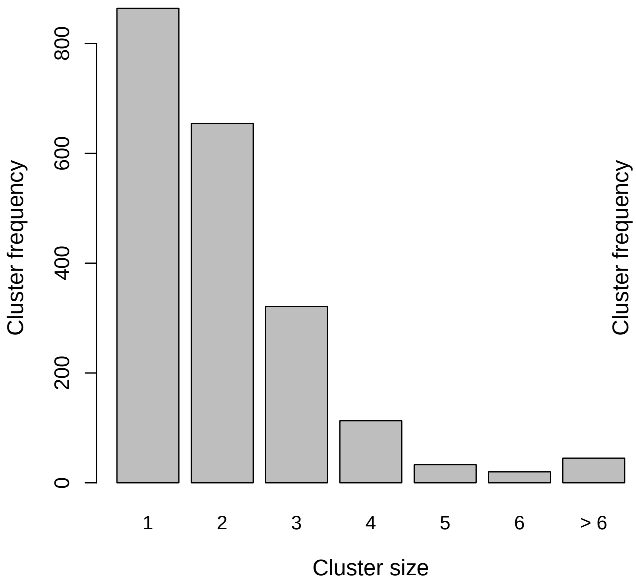

(a) NF-YA

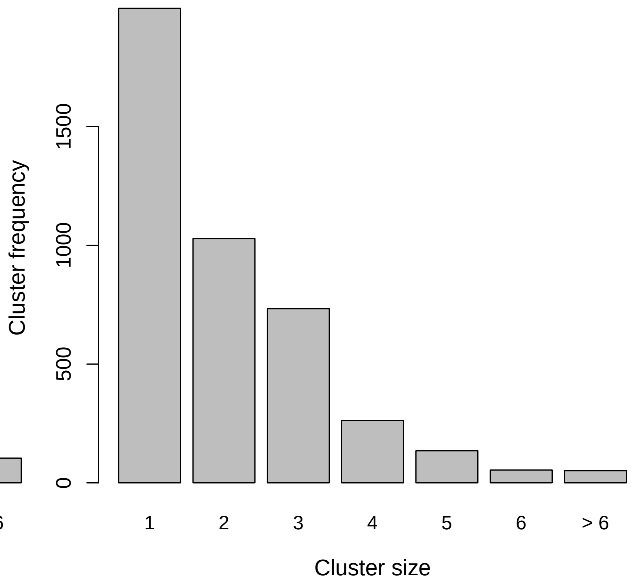

(b) STAT5

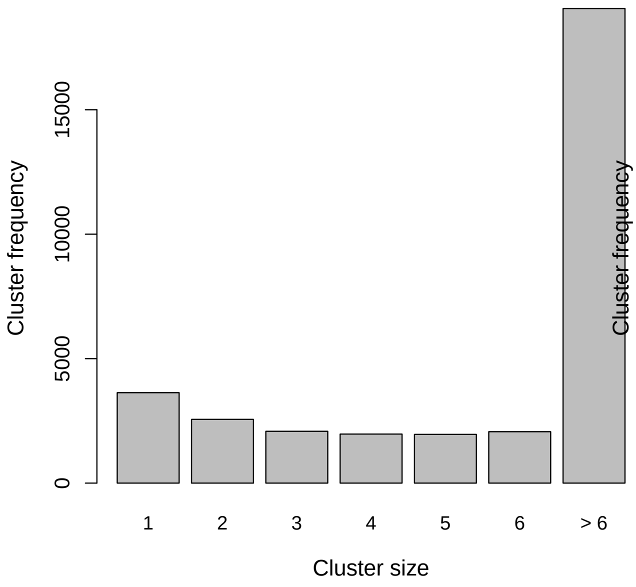

(c) H3K4me3

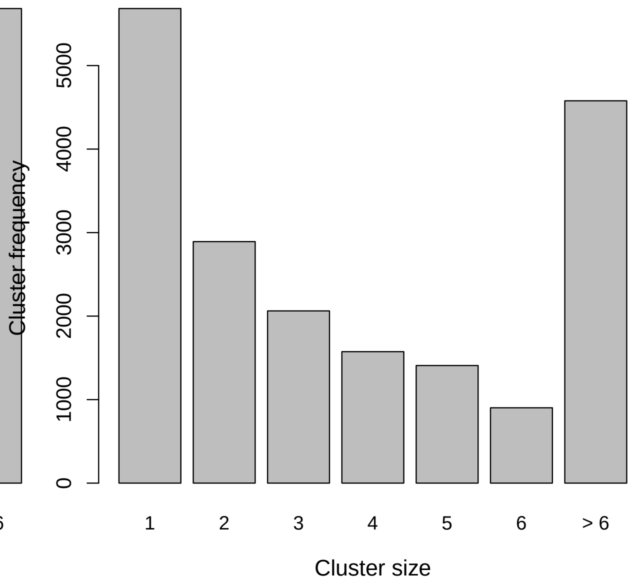

(d) H3ac

Figure S6: Distribution of cluster sizes assembled from detected DB windows in the sliding window method, for each tested ChIP-seq dataset. Cluster sizes refer to the number of windows in each cluster.

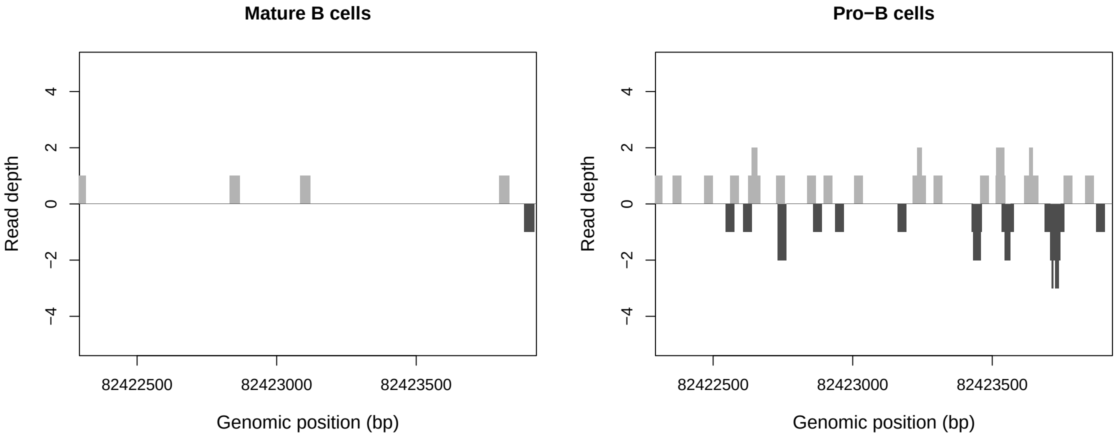

(a) Chromosome 11

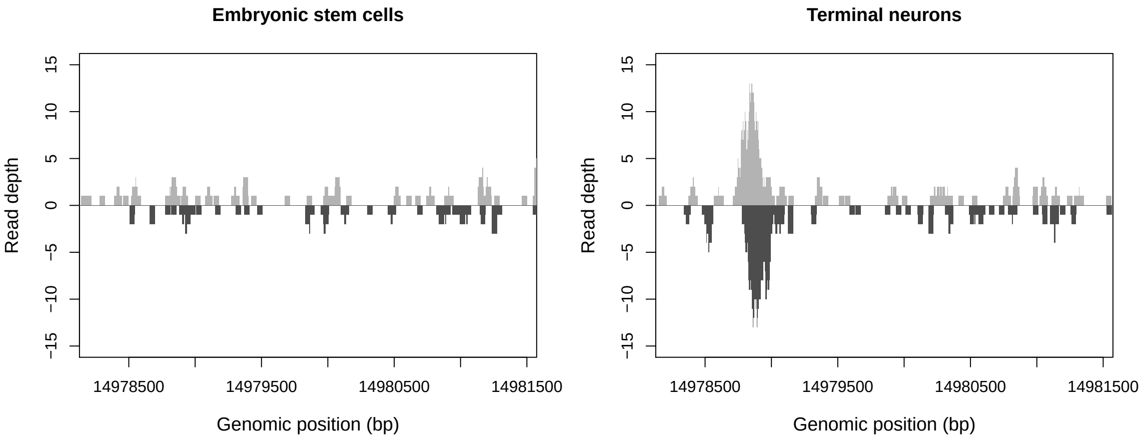

(b) Chromosome 17

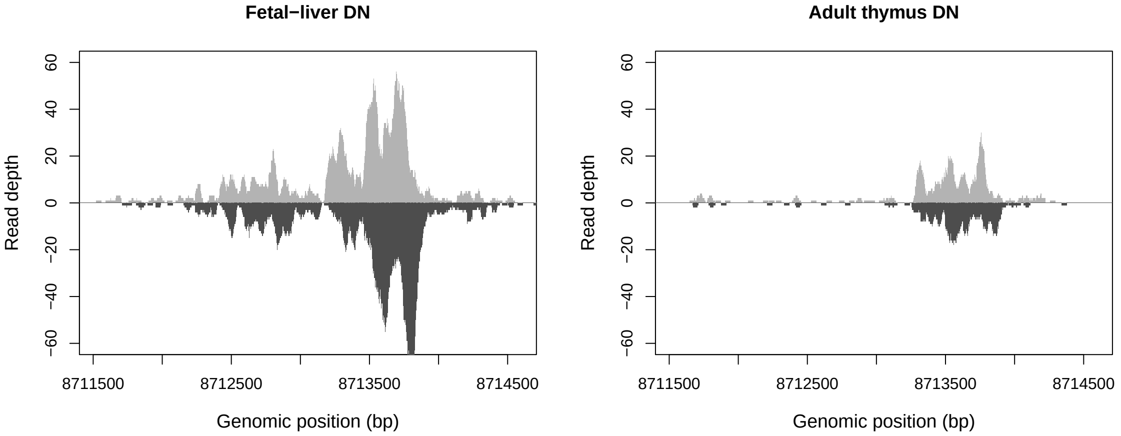

(c) Chromosome 19

Figure S7: Tracks of DB regions detected only by (a) the peak-based method in the H3K4me3 dataset or the hybrid approach in (b) the NF-YA dataset and (c) the H3ac dataset. Positive and negative read depths refer to reads mapped on the forward and reverse strands, respectively. One replicate is shown for each condition in each dataset. Each genomic interval corresponds to the peak called by MACS. DN: double negative cells.

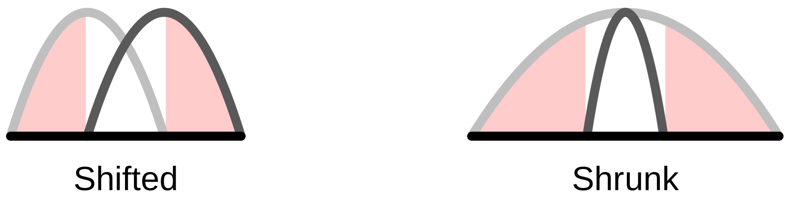

Figure S8: Complex differential binding events in histone mark data. The light grey line represents the peak in one group whereas the dark grey line represents the modified peak in the other group. The horizontal black line represents the boundaries of the enriched region. Shaded red areas correspond to intervals of differential binding.
